# Synchronous regression of nontarget hepatic hemangiomas following direct sclerotherapy of a symptomatic giant hemangioma: A report of two cases

**DOI:** 10.1016/j.radcr.2026.07.013

**Published:** 2026-07-24

**Authors:** Hadi Koohi, Faeze Salahshour, Omid Ghaemi, Hadi Rokni Yazdi, Niloofar Ayoobi Yazdi

**Affiliations:** Department of Radiology, Advanced Diagnostic and Interventional Radiology Research Center (ADIR), Imam Khomeini Hospital Complex (IKHC), Tehran University of Medical Sciences, Tehran, Iran

**Keywords:** Direct sclerotherapy, Nontarget hepatic hemangioma, Synchronous regression

## Abstract

Hepatic hemangiomas are the most common benign mesenchymal tumors of the liver. Occasionally, they can appear as giant hepatic hemangiomas with a diameter greater than 5 cm, causing symptoms such as abdominal pain. Giant hepatic hemangiomas can be treated via surgery or minimally invasive procedures like transarterial chemoembolization or the more recently introduced direct sclerotherapy. We report 2 cases of symptomatic giant hepatic hemangiomas that were successfully treated with percutaneous direct sclerotherapy using bleomycin-lipiodol, which unexpectedly resulted in synchronous multifocal regression of other nontargeted hemangiomas in different liver segments. These 2 cases highlight that direct percutaneous sclerotherapy effectively treats giant hepatic hemangiomas and occasionally may induce the unexpected synchronous regression of untreated, nontarget lesions. This phenomenon suggests potential systemic or inflammatory mechanisms and raises the hypothesis that targeting a primary lesion might influence remote hemangiomas, warranting further prospective investigation.

## Introduction

Hepatic hemangiomas (HH) are the most common benign mesenchymal tumors of the liver, composed of clusters of blood-filled vascular spaces lined by endothelial cells [1,2]. Most are small, asymptomatic, and discovered incidentally. Estimates of prevalence in autopsy series range from approximately 0.4%-7.3% [3]. However, a subset of these lesions, often termed “giant hemangiomas” (typically defined as greater than 5 cm), can become symptomatic due to mass effect, causing abdominal pain, discomfort, or, rarely, complications such as rupture or Kasabach–Merritt syndrome [3,4]. Due to their vascular nature, hemangiomas are predominantly fed by the hepatic artery, a critical characteristic that informs interventional therapeutic strategies [2].

The management of symptomatic or enlarging hepatic hemangiomas remains a subject of debate. Historically, surgical resection or enucleation was the standard treatment, but these procedures are associated with considerable risks of morbidity and hemorrhage, particularly for large or centrally located lesions [5,6]. Consequently, minimally invasive endovascular approaches, such as transarterial embolization (TAE) and transarterial chemoembolization (TACE), have emerged as effective and safer alternatives [7]. TACE, which involves the selective intra-arterial administration of a chemotherapeutic agent (e.g., bleomycin or pingyangmycin) mixed with a lipiodol emulsion and is often followed by embolization, aims to achieve volume reduction by simultaneously delivering cytotoxic action to the endothelial cells and inducing ischemia by blocking the blood supply [8,9]. Numerous studies have demonstrated that TACE is a safe and effective technique, resulting in significant tumor shrinkage and symptom resolution in the treated (target) hemangioma [10,11].

Recently, direct sclerotherapy (DS) of giant symptomatic hepatic hemangiomas was introduced by Rokni et al. with comparable or better size reduction and symptom relief, using a more feasible technique [12,13]. We present 2 patients who experienced significant size reduction in separate, coexisting, nontarget hemangiomas following direct sclerotherapy of a giant target hemangioma. This unexpected observation suggests a possible systemic, humoral, or generalized ischemic effect extending beyond the local effects of sclerotherapy.

## Case presentation

Between 2018 and 2020, we performed DS on 28 patients with symptomatic giant hepatic hemangiomas, followed by a subsequent 2-year follow-up period. Among these individuals, we observed a significant size reduction in nontargeted hemangiomas in 2 patients. Both of these patients had initially presented with classic mass-effect symptoms, including abdominal pain, discomfort, and early satiety, which necessitated therapeutic intervention. (Table 1)

**Table 1:** Demographic data of the patients.

Case	Age/sex	6-mo postprocedural volume of the target lesions	Number of nontarget lesions	Largest nontarget lesion volume and location
Case 1	47 y/o Male	391 mL	3	40 mL in segment 2
Case 2	34 y/o Female	735 mL	2	528 mL in segment 2 and 3

The target lesion in both cases was located in the posterior segments of the right hepatic lobe (segments 6 and 7). All nontarget lesions were confirmed as hepatic hemangiomas on baseline imaging; however, because the pretreatment imaging studies were performed at an outside institution, the raw baseline data were unavailable during the preparation of this manuscript.

### Direct sclerotherapy procedure

Both patients underwent the same standard bleomycin–lipiodol emulsion (B/LE) DS procedure [14], which was performed by an experienced interventional radiologist.

Before the procedure, each patient received prophylactic medication consisting of an intramuscular injection of betamethasone (40 mg/mL depo-medrol) to prevent allergic reactions and an intravenous dose of cefazolin (1 g) to minimize infection risk. Local anesthesia was administered using 10 mL of 2% lidocaine, and conscious sedation was induced with midazolam (1 mg) and fentanyl (100 µg), followed by maintenance with ketamine (5 mg) and propofol (10 mg).

The hemangioma was accessed through normal hepatic tissue using a 22-gauge Chiba needle under ultrasound guidance. Following insertion, 10-20 mL of iodinated contrast (Visipaque) was injected under fluoroscopy to confirm that the lesion was isolated and had no communication with venous, portal, or biliary structures; the needle was repositioned if any vascular or biliary connections were detected. Treatment involved injecting a mixture of 45 IU of bleomycin (diluted in 10 mL of distilled water) and 10 mL of Lipiodol. This emulsion was prepared using a 3-way stopcock between 2 syringes and was administered under fluoroscopic control over 20-30 seconds.

Following therapy, patients were monitored for 6 hours for potential complications such as hemorrhage, pneumonitis, or hypersensitivity reactions. Discharge was permitted for stable patients who exhibited no pain, respiratory issues, or ultrasonographic signs of bleeding. The following day, liver function tests, including aspartate aminotransferase, alanine aminotransferase, alkaline phosphatase, and bilirubin, were conducted. At a 5-month follow-up, patients were reassessed for abdominal or respiratory symptoms, and both hepatic and pulmonary evaluations were repeated to identify any treatment-related toxicity.

### Imaging surveillance protocol

Postprocedure follow-up was conducted for more than 2 years, beginning 6 months after the initial injection. Contrast-enhanced imaging protocols were utilized to assess therapeutic response and monitor all hepatic lesions. Follow-up evaluations consisted of nonenhanced and dynamic extracellular contrast-enhanced liver magnetic resonance imaging (MRI) for Case 1, and 4-phase computed tomography (CT) scans for Case 2.

The standard imaging protocol for the MRI included noncontrast sequences (T1-weighted, T2-weighted, and diffusion-weighted imaging [DWI]) followed by dynamic postcontrast sequences obtained every 30 seconds for up to 3 minutes. The CT protocol included an unenhanced scan followed by contrast-enhanced phases acquired at 30, 60, and 180 seconds after contrast injection.

### Outcome measurement

The volumes of all target and nontarget lesions were calculated using manual segmentation-based volumetry using 3D Slicer image computing platform. Volumetric changes were systematically tracked for both the treated target lesion and the coexisting nontarget hemangiomas to evaluate the overall therapeutic response. Although different imaging modalities were used between the 2 patients (MRI for Case 1 and CT for Case 2), the specific modality was kept consistent for each individual patient across all serial follow-up examinations. Both multiphase CT and dynamic contrast-enhanced MRI are highly reliable for cross-sectional volumetry; maintaining intrapatient modality consistency ensured that the longitudinal volume tracking and calculated percentage reductions remained internally valid and free from cross-modality measurement bias.

### Quantitative analysis of lesion regression

Postprocedural surveillance demonstrated a consistent and significant reduction in lesion volume for both the targeted giant hemangiomas and the coexisting nontarget lesions in both patients.

### Case 1

**Fig. 1:**
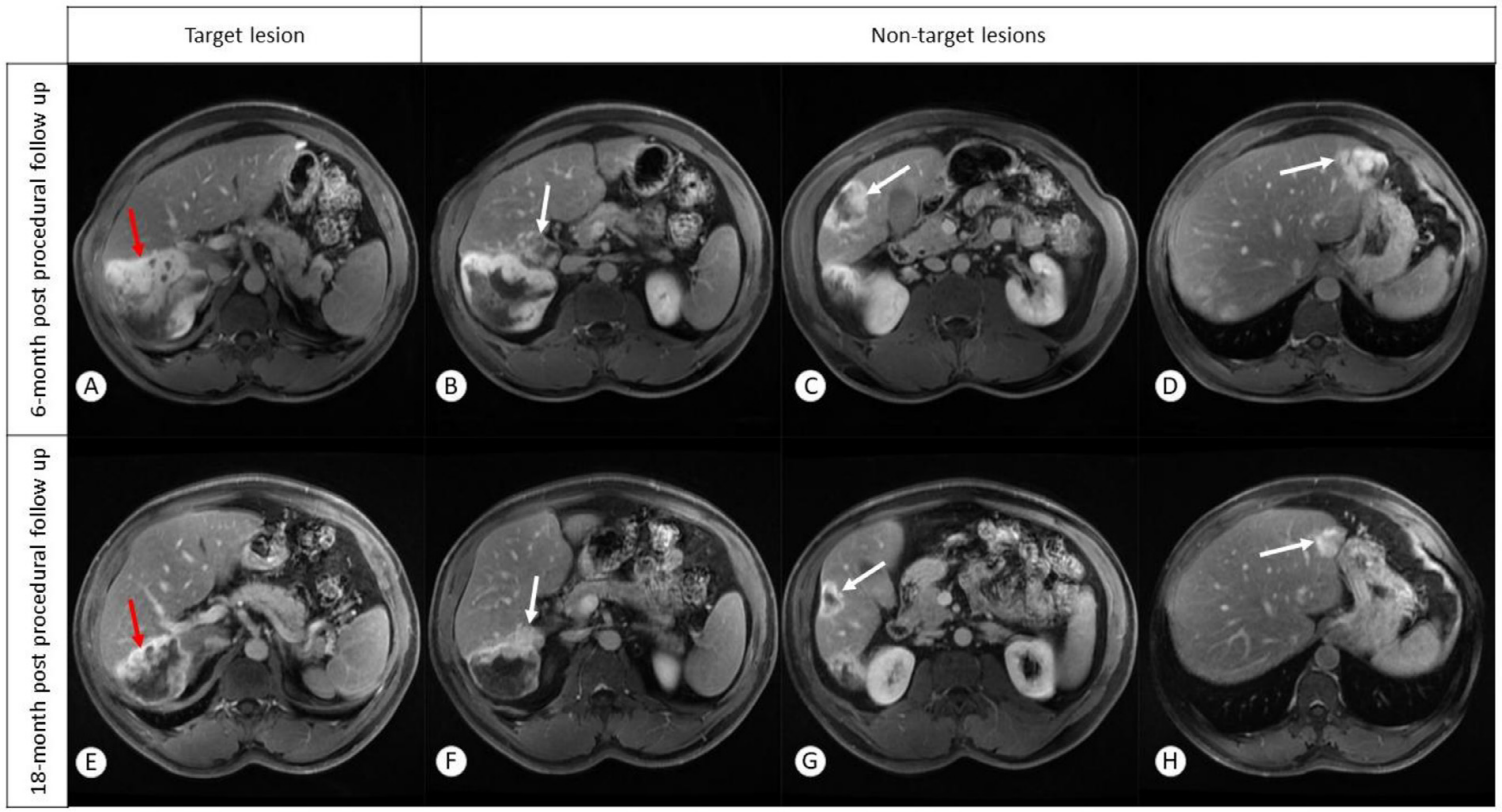
Dynamic MRI of the Case 1 in portal venous phase, showing size reduction of target and nontarget hepatic hemangiomas after direct sclerotherapy. The upper row depicts the 6-month post-DS follow-up and the lower row shows the corresponding changes for each lesion at 18-month post-DS follow-up. The target lesion (red arrows in A and E) shows a significant decrease in size, as well as 3 other nontarget hemangiomas (white arrows).

In the first case (a 47-year-old male), the dominant target lesion located in the posterior segments (segments 6 and 7) demonstrated a volume reduction from a baseline of 391-169 mL at the initial follow-up, continuing to decline to 133 mL at 21 months. This represents a total volume reduction of approximately 66% (Table 2 and Fig. 1).

**Table 2:** Detailed volume changes of target and nontarget hepatic hemangiomas in Case 1.

Case 1	Location (segment)	Lesion’s volume in postprocedural follow-ups			
		6 mo	12 mo	18 mo	27 mo
Lesion 1 (target)	6 and 7	391 mL	169 mL	141 mL	133 mL
Lesion 2	6	23 mL	6 mL	4 mL	3 mL
Lesion 3	6	34 mL	9 mL	6 mL	5 mL
Lesion 4	2	40 mL	12 mL	11 mL	7 mL

Remarkably, the 3 nontarget lesions that were not subjected to direct sclerotherapy exhibited even more pronounced relative shrinkage:

• Lesion 2 (segment 6): Regressed from 23 mL to 3 mL (an approximate 87% reduction).
• Lesion 3 (segment 6): Regressed from 34 mL to 5 mL (an approximate 85% reduction).
• Lesion 4 (segment 2): Regressed from 40 mL to 7 mL (an approximate 82.5% reduction).

As illustrated in Figure 1, the visual radiological evidence correlates closely with the volumetric data, showing a clear decrease in the size of the primary target lesion (panels A and E) alongside the synchronous regression of the 3 nontarget hemangiomas.

### Case 2

**Fig. 2:**
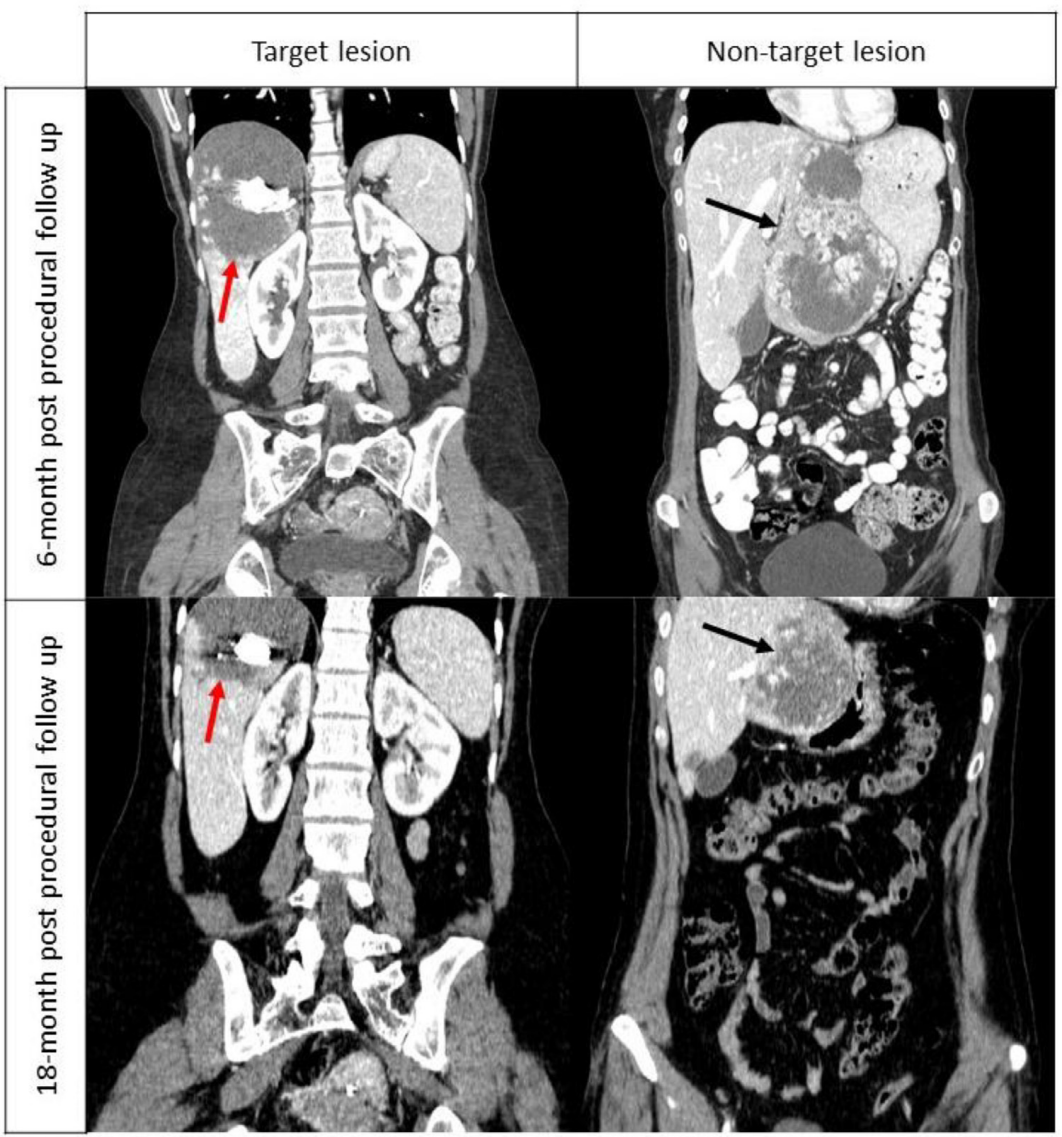
Multiphasic CT scan of the Case 2 in portal venous phase showing significant reduction in size of both target (red arrows) and nontarget (black arrows) hepatic hemangiomas, at 6-month (upper row) and 18-month (lower row) post-DS follow-ups (smallest lesion not shown).

In the second case (a 34-year-old female), the target lesion (segments 6 and 7) measured 735 mL at baseline. Over a 29-month follow-up period, this lesion shrank to 165 mL, achieving a total volume reduction of approximately 77% (Table 3 and Fig. 2).

**Table 3:** Detailed volume changes of target and nontarget hepatic hemangiomas in Case 2.

Case 2	Location (segment)	Lesion’s volume in postprocedural follow-ups			
		6 mo	12 mo	18 mo	29 mo
Lesion 1 (target)	6 and 7	735 mL	210 mL	187 mL	165 mL
Lesion 2	2 and 3	528 mL	165 mL	141 mL	131 mL
Lesion 3	5	8 mL	2 mL	1 mL	1 mL

Similar to Case 1, the nontarget lesions in Case 2 demonstrated a significant size reduction:

• Lesion 2 (segments 2 and 3): This large nontarget lesion decreased from 528 mL to 131 mL, closely mirroring the regression pattern of the treated primary lesion.
• Lesion 3 (segment 5): This smaller lesion reduced from 8 mL to 1 mL.

## Discussion

We report 2 cases of symptomatic giant hepatic hemangiomas that were successfully treated via direct percutaneous sclerotherapy with bleomycin–lipiodol, which unexpectedly resulted in the synchronous multifocal regression of separate, nontargeted, untreated hemangiomas. Traditionally, giant hepatic hemangiomas have been managed with surgical resection or enucleation, which carries substantial risks of morbidity, hemorrhage, and prolonged hospitalization [5,6]. In recent years, minimally invasive interventional techniques have gained attention for the treatment of giant hemangiomas due to their favorable safety profile and efficacy. Among these, direct percutaneous sclerotherapy using bleomycin and ethiodized oil has proven to be a highly effective therapy for hepatic hemangiomas [[12], [13], [14]], causing rapid involution of the vascular spaces by directly exposing the endothelial lining to high concentrations of the sclerosing material. Both of our patients demonstrated marked shrinkage of their targeted giant hemangiomas, a finding consistent with current literature regarding the clinical success, technical feasibility, and safety of this localized interventional approach.

However, the marked regression of separate, noninjected hemangiomas in both patients represents a remarkable and unusual clinical phenomenon. Because percutaneous sclerotherapy is designed to confine the sclerosing agent strictly within the targeted lesion’s vascular bed—thereby minimizing systemic spillage and sparing adjacent hepatic parenchyma—the shrinkage of remote lesions is contrary to standard expectations. In the absence of directly visible, macroscopic circulation between these anatomically separate vascular malformations, this finding suggests that localized percutaneous therapies may exert regional or systemic effects that are not yet fully understood.

The phenomenon of size reduction in nontarget hemangiomas has only recently been reported in a study by Kutlu et al. [15]. In their series, they reported significant volumetric reduction of nontarget hemangiomas after transarterial chemoembolization (TACE) of primary hepatic hemangiomas using a bleomycin–ethiodized oil emulsion. Although their findings mirror our observations, a critical procedural difference exists: Kutlu et al. [15] evaluated patients treated via a transarterial approach, whereas our study utilized direct percutaneous sclerotherapy. Transarterial therapies infuse chemotherapeutic and embolic agents directly into the hepatic arterial tree, which inherently carries a higher potential for regional drug distribution and subsequent exposure of other hepatic segments to the agent. In contrast, our study demonstrates that an isolated, direct percutaneous intralesional injection—which completely bypasses the main arterial tree—can potentially induce the same multifocal volume reduction. This suggests that the observed shrinkage of nontarget hemangiomas may not be solely a consequence of intra-arterial drug redistribution.

Currently, the exact pathophysiology behind this nontarget phenomenon remains entirely unknown, and without concrete data, we can only speculate on potential underlying causes. Previously, it has been shown that systemic chemotherapy for nonhepatic malignancies (such as germ cell tumors) can induce the regression of coexisting hepatic hemangiomas [16]. Even with careful intralesional injection, it is theoretically possible that microscopic venous drainage from a giant hemangioma could lead to gradual systemic absorption. This might introduce subclinical levels of bleomycin into the systemic or hepatic circulation, conceivably exerting a generalized antiangiogenic and sclerosing effect on the susceptible endothelium of nontarget hemangiomas. Another preliminary hypothesis is an underlying immunological or humoral pathway [17]. The localized necrosis and inflammatory response triggered within the target lesion might release cytokines or antiangiogenic factors, inducing an “abscopal-like” regression of structurally identical lesions elsewhere in the liver, as it has been observed in radiation therapy and immunotherapy [18]. However, its relevance in this context is still uncertain. Future studies incorporating postsclerotherapy pharmacokinetic tracking of systemic bleomycin or serum immune markers could determine the hypothetical systemic drug spillover or inflammatory responses.

Our observations highlight clear gaps in the current understanding of the incidence, mechanism, and clinical significance of this nontarget phenomenon during percutaneous interventions, necessitating further prospective investigation.

This study is primarily limited by the lack of baseline pretreatment imaging from referring institutions. Although these lesions were initially diagnosed as hepatic hemangiomas, the absence of original imaging prevents independent verification of their baseline characteristics, count, and size at this point of time. Consequently, while serial postprocedural imaging confirmed significant volumetric reduction of the nontarget lesions, the lack of raw baseline data may limit our ability to analyze pretreatment growth kinetics or rule out prior spontaneous regression.

## Conclusion

Direct percutaneous sclerotherapy with a bleomycin–lipiodol emulsion is a highly effective, minimally invasive treatment for symptomatic giant hepatic hemangiomas, providing excellent volume reduction and symptom relief. Our report highlights that this localized therapy can also induce the synchronous regression of separate, untreated hemangiomas within the same patient. The occurrence of this phenomenon following direct percutaneous injection—contrasting with recently reported transarterial data—suggests the presence of complex, yet currently unproven, underlying mechanisms, such as minor systemic drug absorption or a generalized inflammatory response. This nontarget effect raises an interesting hypothesis: in certain patients with multifocal symptomatic hemangiomas, the treatment of the primary dominant lesion might occasionally influence the untreated remote lesions. Future prospective studies utilizing postprocedural pharmacokinetic tracking of systemic bleomycin levels and serial monitoring of serum inflammatory markers are warranted to unravel the exact pathophysiology of this phenomenon and determine if this hypothesis holds true in larger cohorts.

## Declaration of generative AI and AI-assisted technologies in the manuscript preparation process

During the preparation of this work, the authors used LLMs to check the grammar and fluency of the text. After using this tool/service, the authors reviewed and edited the content as needed and take full responsibility for the content of the published article.

## Ethics approval

This report has been performed in accordance with the Declaration of Helsinki.

## Patient consent

Written informed consents were obtained from the patient for publication of this case report and any accompanying images.
